# Supplementary material for: Gene-Function-Based Clusters Explore Intricate Networks of Gene Expression of Circulating Tumor Cells in Patients with Colorectal Cancer
Source: Biomedicines. 2023 Jan 6;11(1):145. doi: 10.3390/biomedicines11010145 (PMC9855519; doi:10.3390/biomedicines11010145)

Supplementary Figure S1. STRING Analysis of 15 Investigated Genes

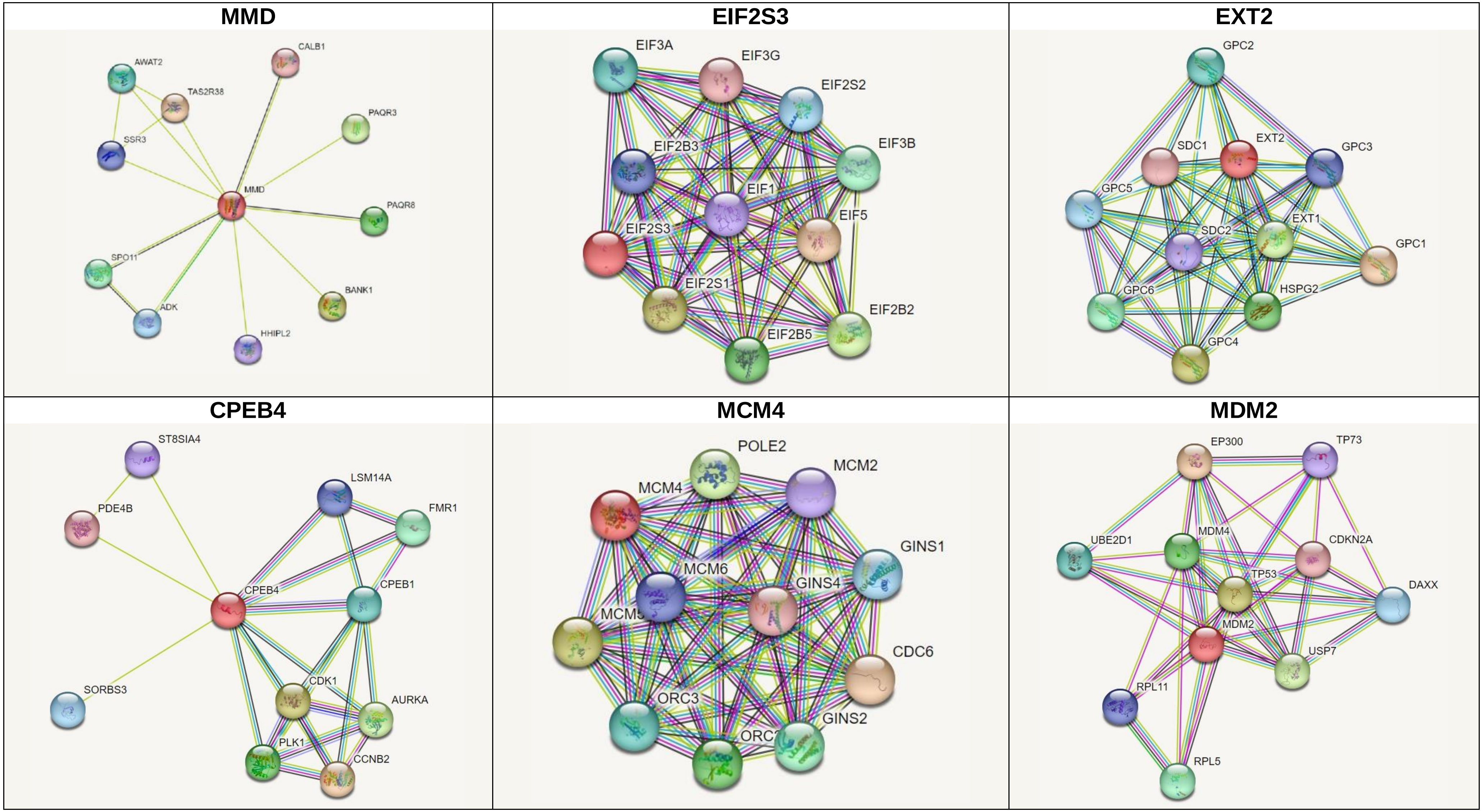

Supplementary Figure S1. STRING Analysis of 15 Investigated Genes (continued)

WEE1

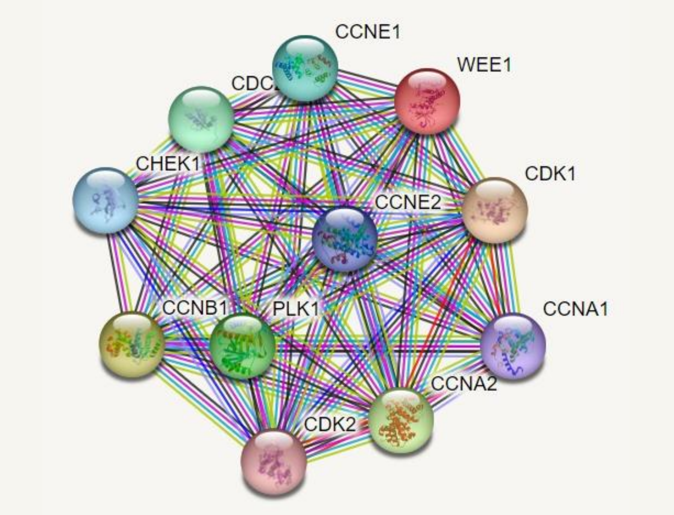

POLDIP2

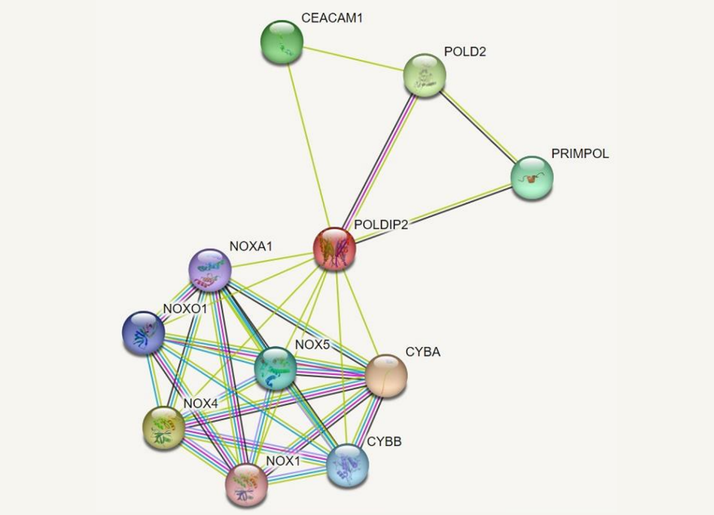

ZNF264

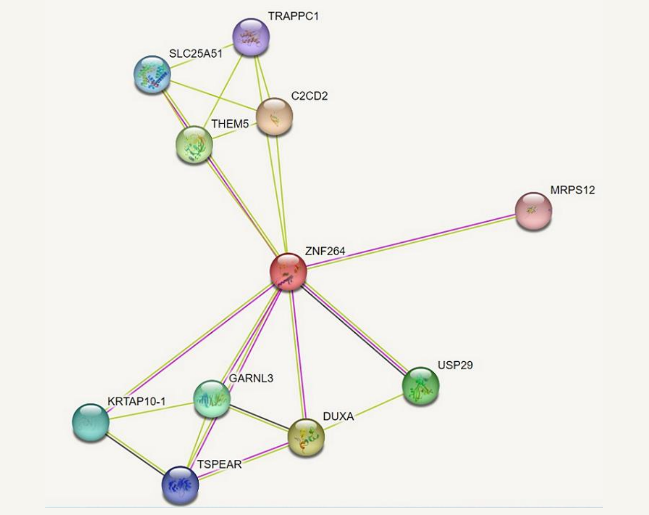

RNF4

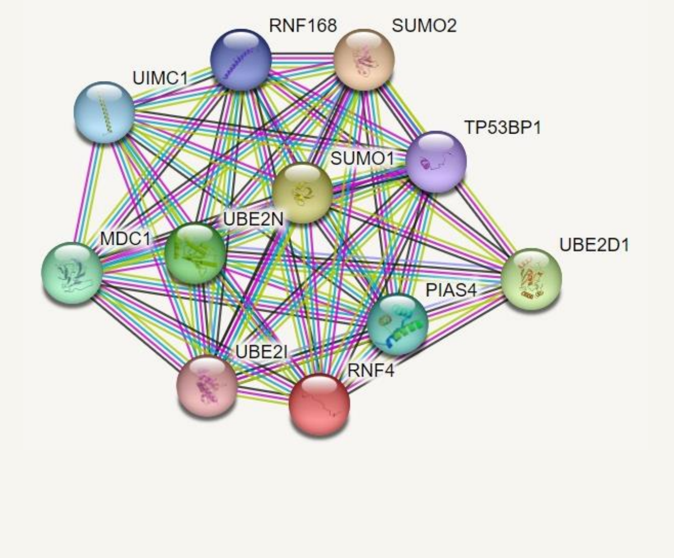

IRF4

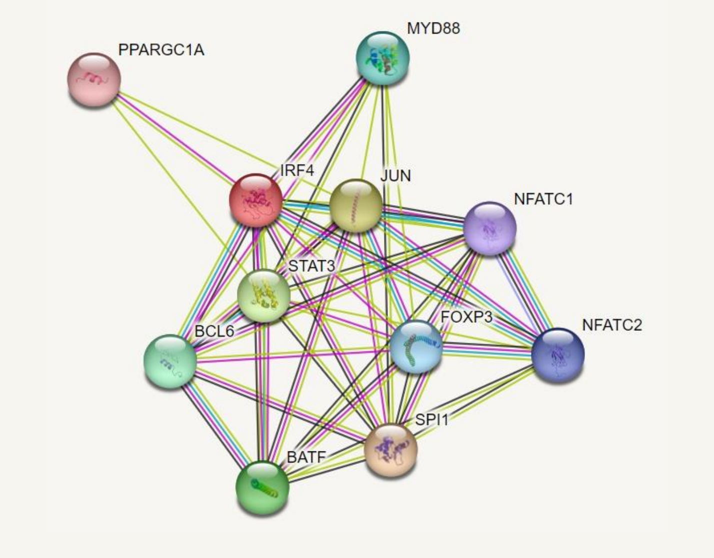

STAT2

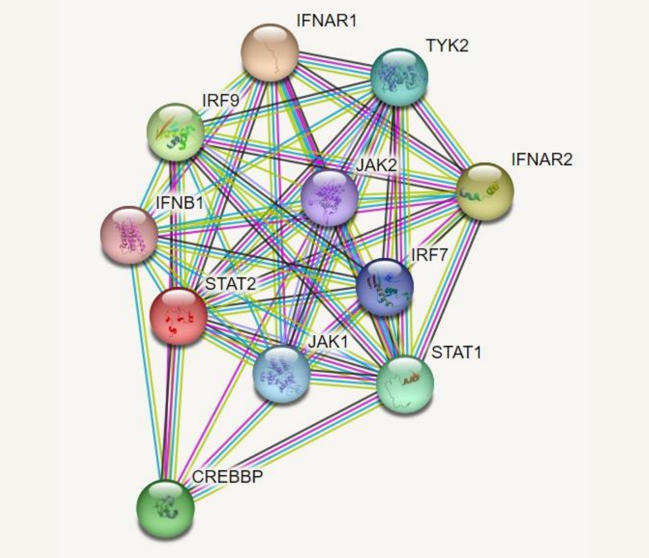

Supplementary Figure S1. STRING Analysis of 15 Investigated Genes (continued)

GRB2

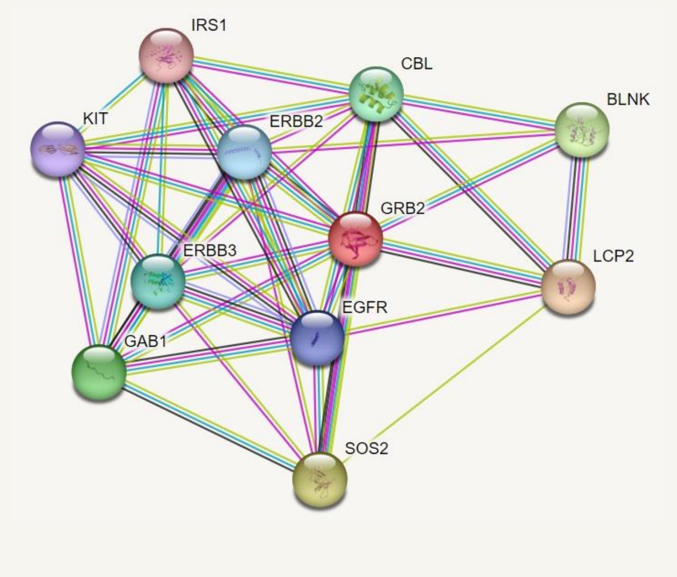

NF1

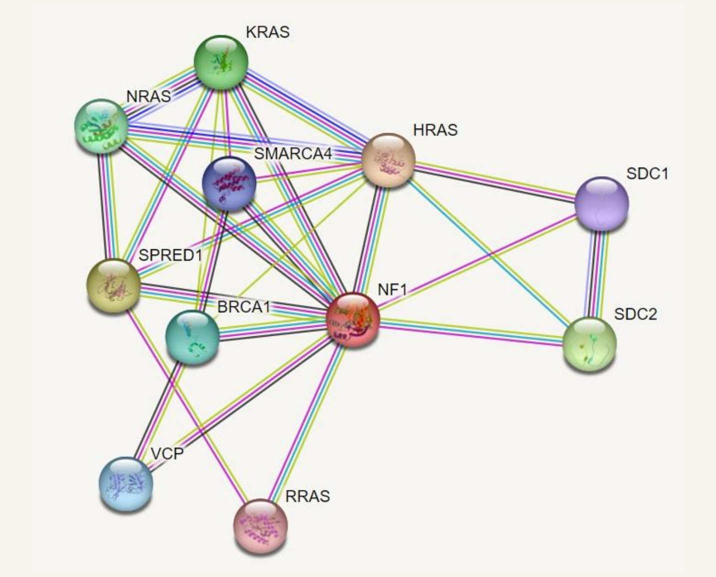

DUSP6

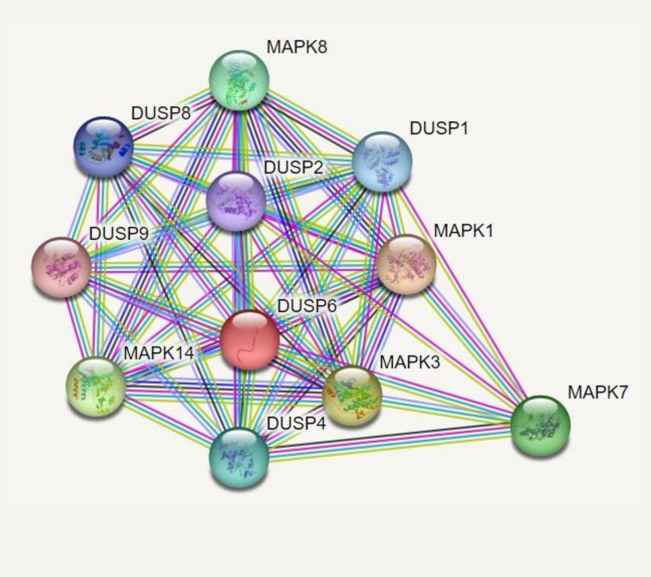

Supplement: Supplementary file 1 [file biomedicines-11-00145-s001.zip › biomedicines-2076112-Supplementary Figure S1.pdf]
