# Supplementary material for: Gene-Function-Based Clusters Explore Intricate Networks of Gene Expression of Circulating Tumor Cells in Patients with Colorectal Cancer
Source: Biomedicines. 2023 Jan 6;11(1):145. doi: 10.3390/biomedicines11010145 (PMC9855519; doi:10.3390/biomedicines11010145)
Supplement: Supplementary file 1 [file biomedicines-11-00145-s001.zip › biomedicines-2076112-Supplementary TableS1.pdf]

Gene function-based clusters explore intricate networks of gene expressions in patients with colorectal cancer

Chi-Shuan Huang, Harn-Jing Terng, Yi-Ting Hwang

Supplementary Tables

Supplementary Table S1A

Logistic analysis for gene-cluster interactions. Percent of significance (PS) of each cluster-grouped gene for multiple cluster models are listed.

Model ID	Combination	*MMD*	*EIF2S3*	*EXT2*	*CPEB4*	*MCM4*	*MDM2*	*WEE1*	*POLDIP2*	*ZNF264*	*RNF4*	*IRF4*	*STAT2*	*GRB2*	*NF1*	*DUSP6*
**Two-cluster models**																
M2	LY/CY	1.0				96.0	100.0	99.8	0.0							
M3	LY/TRf	29.6								93.8	19.4					
M4	LY/TRm	5.2										0.0	21.0			
M9	TLA/TRf		100.0	90.0	100.0					54.8	0.4					
M10	TLA/TRm		99.8	94.6	100.0							0.8	2.0			
M13	TLA/CY		100.0	99.8	69.0	17.0	100.0	1.0	18.2							
M15	LY/TLA	1.2	100.0	81.8	100.0											
M22	LY/SN	0.0												0.0	0.6	100.0
M23	CY/SN					87.4	100.0	11.6	6.8					0.0	71.6	99.4
M24	TLA/SN		99.8	97.4	100.0									21.0	1.2	99.8
M25	TRf/SN									84.0	76.2			6.8	23.4	100.0
M26	TRm/SN											0.6	2.2	0.0	3.0	100.0
M38	CY/TRf					99.8	100.0	96.4	0.6	2.2	98.8					
M39	CY/TRm					98.2	100.0	96.0	0.0			0.0	0.0			
M86	TRf/TRm									69.8	73.2	0.0	65.2			

Supplementary Table S1A (continue)

Model ID	Combination	*MMD*	*EIF2S3*	*EXT2*	*CPEB4*	*MCM4*	*MDM2*	*WEE1*	*POLDIP2*	*ZNF264*	*RNF4*	*IRF4*	*STAT2*	*GRB2*	*NF1*	*DUSP6*
**Three-cluster models**																
M5	LY/CY/SN	0.0				87.2	100.0	8.6	7.8					0.0	69.4	99.2
M6	LY/TRf/SN	0.0								76.2	78.0			7.0	13.4	100.0
M7	LY/TRm/SN	0.2										0.8	2.2	0.0	1.2	100.0
M11	TLA/TRf/SN		92.4	97.2	100.0					84.4	1.2			42.0	20.0	99.8
M12	TLA/TRm/SN		100.0	95.6	100.0							11.4	30.6	40.6	1.8	100.0
M14	TLA/CY/SN		100.0	99.8	79.6	9.0	100.0	0.4	0.0					2.0	30.8	99.0
M16	LY/TLA/CY	1.0	100.0	99.8	77.8	15.4	100.0	0.2	21.2							
M18	LY/TLA/TRf	0.0	100.0	90.2	100.0					46.0	0.4					
M19	LY/TLA/TRm	2.4	99.4	95.4	100.0							0.2	6.0			
M28	LY/TLA/SN	100.0	96.4	100.0	100.0									81.4	0.2	100.0
M40	LY/CY/TRf	0.0				99.2	100.0	96.0	0.2	3.2	98.8					
M42	LY/CY/TRm	1.2				95.6	100.0	97.8	0.0			0.0	0.0			
M87	TLA/CY/TRm		100.0	99.6	71.0	9.8	100.0	3.4	26.6			1.0	0.6			
M88	TLA/CY/TRf		100.0	77.6	38.0	29.0	100.0	1.8	24.0	5.0	53.0					
**Four-cluster models**																
M17	LY/TLA/CY/SN	96.4	99.8	100.0	99.6	7.0	100.0	4.6	0.0					40.6	13.4	100.0
M20	LY/TLA/TRf/SN	94.6	90.6	97.2	100.0					19.6	2.0			84.0	3.0	100.0
M21	LY/TLA/TRm/SN	100.0	99.4	99.6	100.0							9.0	63.0	91.6	0.6	100.0
M41	LY/CY/TRf/SN	1.0				97.4	100.0	0.2	1.4	32.4	100.0			3.6	98.8	98.2
M43	LY/CY/TRm/SN	0.0				96.2	100.0	6.2	6.6			0.0	37.2	0.0	78.2	100.0
M85	TLA/CY/TRf/SN		99.0	91.2	65.8	9.4	100.0	0.4	0.0	69.6	69.0			18.6	84.0	97.4
M105	TLA/CY/TRm/SN		100.0	99.8	77.4	3.8	100.0	0.2	0.0			1.0	43.6	6.6	39.6	99.6

Supplementary Table S1B

Logistic analysis for cluster-cluster interactions. Odds ratio (OR) of each cluster-grouped gene for multiple cluster models are listed.

Model ID	Combination	*MMD*	*EIF2S3*	*EXT2*	*CPEB4*	*MCM4*	*MDM2*	*WEE1*	*POLDIP2*	*ZNF264*	*RNF4*	*IRF4*	*STAT2*	*GRB2*	*NF1*	*DUSP6*
**Two-cluster models**																
M2	LY/CY	1.14				2.09	5.87	0.40	0.92							
M3	LY/TRf	1.23								1.95	0.66					
M4	LY/TRm	1.16										1.04	1.42			
M9	TLA/TRf		0.32	0.40	2.87					1.80	1.02					
M10	TLA/TRm		0.33	0.46	2.87							1.17	1.36			
M13	TLA/CY		0.19	0.25	1.72	1.58	8.66	0.77	1.62							
M15	LY/TLA	0.85	0.34	0.55	3.43											
M22	LY/SN	0.90												1.10	0.82	2.76
M23	CY/SN					2.00	6.01	0.66	0.64					1.00	0.42	2.19
M24	TLA/SN		0.30	0.35	2.82									1.65	0.74	2.35
M25	TRf/SN									2.06	0.46			1.32	0.58	2.45
M26	TRm/SN											1.19	0.75	1.11	0.74	2.87
M38	CY/TRf					2.45	8.44	0.46	1.21	1.40	0.29					
M39	CY/TRm					2.18	5.95	0.42	0.93			0.98	0.99			
M86	TRf/TRm									1.77	0.47	1.12	1.67			

Supplementary Table S1B (continue)

Model ID	Combination	*MMD*	*EIF2S3*	*EXT2*	*CPEB4*	*MCM4*	*MDM2*	*WEE1*	*POLDIP2*	*ZNF264*	*RNF4*	*IRF4*	*STAT2*	*GRB2*	*NF1*	*DUSP6*
**Three-cluster models**																
M5	LY/CY/SN	0.95				2.03	5.96	0.67	0.63					1.02	0.42	2.24
M6	LY/TRf/SN	0.92								2.00	0.46			1.35	0.61	2.55
M7	LY/TRm/SN	0.89										1.20	0.75	1.13	0.77	3.05
M11	TLA/TRf/SN		0.35	0.23	2.90					2.31	0.80			1.83	0.50	2.26
M12	TLA/TRm/SN		0.25	0.36	2.79							1.41	0.58	1.79	0.70	2.75
M14	TLA/CY/SN		0.21	0.17	1.88	1.51	7.48	1.30	0.93					1.56	0.45	2.20
M16	LY/TLA/CY	0.84	0.19	0.23	1.92	1.55	8.81	0.79	1.66							
M18	LY/TLA/TRf	0.91	0.33	0.40	3.06					1.75	1.01					
M19	LY/TLA/TRm	0.83	0.35	0.44	3.26							1.15	1.41			
M28	LY/TLA/SN	0.49	0.32	0.23	4.33									2.26	0.86	3.01
M40	LY/CY/TRm	1.04				2.41	8.36	0.45	1.20	1.42	0.29					
M42	LY/CY/TRm	1.15				2.11	5.95	0.40	0.93			0.98	0.97			
M87	TLA/CY/TRm		0.18	0.25	1.74	1.52	9.05	0.73	1.71			1.19	0.83			
M88	TLA/CY/TRf		0.18	0.33	1.58	1.79	10.62	0.74	1.69	1.46	0.41					
**Four-cluster models**																
M17	LY/TLA/CY/SN	0.51	0.21	0.11	2.73	1.49	6.81	1.59	0.80					2.17	0.51	2.87
M20	LY/TLA/TRf/SN	0.53	0.35	0.20	4.17					1.74	0.75			2.39	0.66	2.80
M21	LY/TLA/TRm/SN	0.46	0.25	0.25	4.45							1.40	0.48	2.56	0.82	3.82
M41	LY/CY/TRf/SN	0.84				2.34	10.18	0.92	0.70	1.86	0.18			1.44	0.25	2.22
M43	LY/CY/TRm/SN	0.91				2.20	6.69	0.70	0.62			1.00	0.56	1.19	0.39	2.80
M85	TLA/CY/TRf/SN		0.22	0.21	1.84	1.58	9.86	1.32	0.85	2.39	0.33			1.83	0.27	2.14
M105	TLA/CY/TRm/SN		0.17	0.19	1.88	1.48	8.21	1.28	1.03			1.24	0.50	1.67	0.44	2.62
